# INVITED PAPERS

**DOI:** 10.1038/sj.bjc.6601053

**Published:** 2003-06-27


**British Journal of Cancer (2003) 88**(Suppl 1), S1 – S8
© 2003 Cancer Research UK All rights reserved 0007 – 0920/03 $25.00
**www.bjcancer.com**

### INVITED PAPERS

### S1

### STRUCTURE, DYNAMICS AND ENERGETICS IN ANTISENSE AND ANTIGENE OLIGONUCLEOTIDES

Andrew N. Lane, James Graham Brown Cancer Center,
University of Louisville, KY, USA.

The development of anticancer drugs is a complex process involving many, often competing, factors. We have been working on the structural and energetic analysis of targeting both DNA and mRNA by oligonucleotides. Some of the major problems are ones of thermodynamic and biochemical stability, uptake and intracellular delivery. In a long-standing collaboration with T. Brown (University of Southampton) we have examined the structures, thermodynamics and dynamics of several DNA. RNA hybrid duplexes (antisense), and the corresponding DNA and RNA duplexes. The origin of the stability of the duplexes, and their solution properties are not intuitively obvious, and require a somewhat different way of dealing with the problem (1). In particular the roles of conformational flexibility and hydration will be emphasized. The stabilisation by particular base modifications has also been characterised. In parallel we have also been investigating the thermodynamic stability and conformations of parallel triple helices (antigene), and the influence of modifications on these properties. The structural and thermodynamics analysis has established some important ground rules for stabilizing these structures (2), which has lead to a new generation of modified nucleotides that have greatly enhanced biochemical and thermodynamic stability. The results underline the importance of collaborations between structural biologists, biophysicists, computational biologists and synthetic chemists.
There are some important cell biological problems remaining that have not yielded to direct conformational and thermodynamic analysis. However, some possibilities for improving the efficacy of antigene approaches will be discussed, and strategies for improving oligonucleotide design based on structural data.

(1) Gyi et al. (1998) *Biochemistry* **37**, 73-80
(2) Asensio et al. (1999) *Structure* **7**, 1-11

### S2

### ENERGETICS AND BIOPHYSICS OF DRUG BINDING FOR CANCER AGENTS – "SMART DRUG DESIGN"

Terry C. Jenkins, YCR Laboratory of Drug Design, Tom Connors Cancer Research Centre, University of Bradford, West Yorkshire BD7 1DP, UK

Rational drug development using template-directed design approaches offers an opportunity to develop novel agents for key biomolecular targets in tumour cells. In this strategy, structural methods are augmented by molecular modelling and biophysical assays, so that it possible to fine-tune a small molecule to achieve exquisite potency. Using this 'molecular tailoring' approach, a novel class of potent DNA cross-linking agents bearing N2(guanine)-alkylating pyrrolobenzodiazepine (PBD) sub-units has been evolved from otherwise inert *bis*-(amidinophenyl) ethers, such as propamidine and pentamidine. The latter drugs act as minor groove-binding agents for double-stranded DNA (A/T-preferential binding), and their molecular platform is eminently suitable as a delivery vehicle for modification (mutation) or attachment of suitable alkylating war-heads. Early success from this approach was realised with DSB-120, the first reported PBD dimer (with Profs. DE Thurston and JA Hartley, and Spirogen colleagues). This compound shows *in vitro* potency (e.g. $IC_{50}$ = 0.25 μM for human K562 leukaemia) and efficient interstrand DNA cross-linking ($C_{50\%}$ = 55 nM towards plasmid pBR322) for 5'-GATC targets in DNA, but lacked *in vivo* efficacy. Further manipulation of the PBD residues resulted in SJG-136, where both cytotoxic potency and reactivity are enhanced ($IC_{50}$ = 0.04 μM for K562; $C_{50\%}$ = 45 nM), and this dimer has now been selected for clinical trial. The design, evaluation and evolution process will be discussed, together with recent (i.e. hotter!) developments for this remarkable class of anti-tumour agent.

### S3

### TOPOISOMERASE I AND CANCER CHEMOTHERAPY : NEW INHIBITORS, NOVEL CONCEPTS

Christian Bailly, INSERM U-524 and Centre Oscar Lambret,
Lille, France (*bailly@lille.inserm.fr*)

Fifteen years of efforts in targeting topoisomerase I for the discovery of anticancer agents have lead to the identification of several families of compounds capable of stabilizing DNA-topoisomerase I covalent complexes. The lead series is the camptothecin family with two drugs, topotecan and irinotecan, approved for cancer treatment and several second (e.g. lurtotecan, exatecan) and third (e.g. diflomotecan) generations of camptothecin analogs currently in clinical trials (1). However, apart from the camptothecins, only a few topoisomerase I poisons have reached phase I clinical trials. Promising results have been reported with glycosyl indolocarbazoles (2) but so far there is still no non-CPT topoisomerase I poisons in advanced clinical trials. Continuous efforts in the optimization of indolocarbazoles, indenoisoquinolines, or benzimidazoles will hopefully lead to effective candidates for clinical development but the need for new series of topoisomerase I poisons remains pressing. Our successful attempts at discovering three series of potent topoisomerase I poisons: lactone-modified camptothecins, glycosyl indolocarbazoles and very recently a new series of marine products, will be presented. In antitumor pharmacology but also in the field of infectious diseases, topoisomerase I remains an actively studied protein which can be targeted via its well known DNA cleavage activity and/or its newly identified kinase activity.

1. Bailly, C. (2003) Homocamptothecins: potent topoisomerase I inhibitors and promising anticancer drugs. *Crit. Rev. Oncol. Hematol. 45*, 91-108.
2. Bailly, C. (2003) Targeting DNA and topoisomerase I with indolocarbazole antitumor agents. In "Small Molecule DNA and RNA Binders", Vol. 2, Eds: Demeunynck M, Bailly C., Wilson WD., Wiley-VCH, pp. 538-575.

### S4

### PATTERN RECOGNITION AND GRID COMPUTING IN DRUG DISCOVERY

W Graham Richards, Department of Chemistry, University of Oxford

The completion of the sequencing of the human genome is being followed by massive investment in producing crystal structures of proteins. Many of these may be targets for anti-cancer drugs. We have developed pattern recognition techniques to find the binding sites on proteins of known structure.
It is then possible to do virtual screening using computational methods. Grid computing where this task is distributed over many machines provides massive power for very extensive searches. By adopting screen saver technology we have reached a stage where we have over two million personal computers performing this task in over 200 countries. This has provided over 200,000 years of CPU time, permitting the screening of some 3.5 billion drug-like molecules against the following protein targets: superoxide dismutase; vascular endothelin growth factor; cyclooxygenase; tyrosine kinase; ras; insulin tyrosine kinase; fibroblast growth factor receptor; CDK-2; protein-tyrosine-phosphatase; farnesyltransferase; raf and VEGFr1.

Fuller details can be found at ww.chem.ox.ac.uk/curecancer.html

### S5

### DRUGS THAT TARGET TUMOUR HYPOXIA: THE PROMISE AND THE CHALLENGE

William A Denny

*Auckland Cancer Society Research Centre, Faculty of Medical & Health Sciences, The University of Auckland, Private Bag 92019, Auckland, New Zealand*

Tumour hypoxia resulting from inefficient blood vessel networks in growing solid tumours is a widespread phenomenon, with about 65% of all human tumours surveyed containing >35% of hypoxic cells. These cells are resistant to radiotherapy and some chemotherapeutic drugs, and up-regulate a variety of survival genes. Although such hypoxia is essentially restricted to solid tumour tissue, and can thus be potentially exploited for selective chemotherapy, this has not proved easy. Four main classes of hypoxia-activated (bioreductive) prodrugs have been described; quinones, nitroaromatic mustards, aliphatic N-oxides and aromatic N-oxides. In the latter category is tirapazamine, which is currently in Phase III trials, and is expected to become the first hypoxia-activated prodrug registered for clinical use.

Experience with each of these classes has revealed necessary design requirements for such prodrugs. These include efficient extravascular diffusion of the prodrug to hypoxic regions, efficient and selective activation to a toxic activated species (effector) by the small proportion of hypoxic cells present, and efficient (albeit controlled) diffusion of the effector to kill surrounding non-hypoxic tumour cells. The latter is governed both by the stability (life-time) of the effector and its lipophilicity.

Measuring these properties, and using the quantitative data generated to design improved prodrugs, will be illustrated by recent work in our laboratory with both nitroaromatic mustards and aromatic N-oxides (analogues of tirapazamine).

### S6

### Understanding how anti-CD20 monoclonal antibodies work. (Bournemouth July 03)

Martin Glennie, Suzanne Morgan, Claude Chan, Ruth French, Alison Tutt, Peter Johnson and Mark Cragg

Of all the 'naked' anti-cancer antibodies that have been investigated to date, those directed at CD20 on B-cell lymphoma appear to be the most active. However, it is still unclear which effector functions are responsible for this potency. CD20 mAb are unusual in that they are capable of mediating multiple effector functions, including antibody dependent cellular cytotoxicity (ADCC), complement dependent cytotoxicity (CDC), and direct transmembrane signalling for apoptosis. Since their ability to induce apoptosis is one of the features that distinguishes them from most other anti-lymphoma mAb, it is generally felt that this property is probably important in vivo. However, it is equally plausible that in vivo activity relies on a combination of effector functions, including the ability to generate an as yet undefined active immune response which may take months to acquire.

We have recently investigated the effector functions of an extended panel of anti-CD20 mAb. Whilst they all mediate ADCC provided they are of the correct isotype, we find that they can be divided into two very distinct sub-groups based on their ability to mediate CDC. Type 1 anti-CD20 mAb, designated Rituximab-like, are highly active in CDC, whilst type 2, designated B1-like are almost completely inactive. The two sub-types appear to be directly determined by the readiness with which they translocate CD20 into lipid 'rafts'. Interestingly, although poor at CDC, the B1-type mAb are generally more potent at apoptosis.

### S7

### DNA VACCINATION IN B CELL MALIGNANCIES AND SOLID TUMOURS

Christian Ottensmeier, Cancer Sciences Division, University of Southampton.

Our improved understanding of the human immune system now allows us to to design rational immunological treatment strategies in the laboratory and to assess their effect in clinical trials. DNA vaccination can be used as a platform technology for the induction of specific anti-tumour immunity. So far our vaccines have been patient specific and encode the tumour derived immunoglobulin heavy and light chain variable region genes in a single chain format (scFv). In order to promote immunity the scFv is linked to a strongly immunostimulatory sequence from fragment C of tetanus toxin (FrC). The vaccine backbone is a plasmid cassette which additionally contains CpG motifs as unspecific immune enhancers.

This vaccine design is being tested in a series of tightly focussed phase I/II clinical trials. The first trial in three centres investigates patients with follicular lymphoma (FL). Consistent induction of immunity against FrC and in some patients against the tumour derived immunoglobulin can be detected. In a parallel study donors of allografts are vaccinated against their siblings' myeloma. Early data suggests induction of significant anti-idiotypic responses.

In solid tumours, anti-tumour immunity requires the induction of CD8+ T cells. In murine system this can be achieved with a modified vaccine design which induces high levels of epitope-specific T cells. These can protect against tumour challenge and act therapeutically. This novel design is now undergoing testing in patients and if successful opens the opportunity of designer vaccination against a wide variety of solid tumour antigens.

### S8

### CELLULAR THERAPIES FOR LEUKEMIA AND LYMPHOMA

Malcolm K. Brenner, Center for Cell and Gene Therapy,
Baylor College of Medicine, Houston, Texas USA

The success of monoclonal antibody therapies for leukemia and lymphoma has invigorated efforts to use the immune system to eradicate these diseases, in the hope that the greater specificity of the system will produce fewer adverse effects than conventional small molecule therapeutics. The ability of donor lymphocyte infusions to eradicate residual host chronic myeloid leukemia (and with lesser frequency AML, lymphoma and myeloma) after allogeneic stem cell transplantation has increased optimism that cellular as well as humoral immune mechanisms may be usefully applied to disease treatment. The field has also been encouraged by recent technological improvements in molecular engineering and gene transfer, which allow the specificity and function of the potential effector cells to be enhanced.

In this presentation I will describe pre-clinical and clinical results for three o[illegible] major approaches currently in use for leukemia and lymphoma. First is the active immunotherapy, in which there is local injection of leukemia or lymphoma cells expressing immunostimulatory genes that trigger a systemic cell mediated immune response against weak or tolerizing tumor associated antigens. This approach is currently being studied in patients with ALL, AML and CLL. Second is the adoptive transfer of tumor antigen specific T cells, an approach we are currently studying in EBV associated malignancies including Hodgkin Disease and Nasopharyngeal cancer. Finally, we are developing chimeric receptor expressing T cells that are specific for tumor associated antigens (such as CD19) via their chimeric receptor, and for persistent viral antigens such as EBV via their native receptor. These chimeric EBV specific T cells receive multiple co-stimulatory signals when they engage their native target which enhances their activity against the malignant target recognized through their chimeric receptor.

These approaches are promising in terms of the immunologic and antitum or responses they produce, and are cost competitive with small molecule based therapies. However, their eventual widespread implementation into clinical practice will require a reallocation of resources by the pharmaceutical industry and by medical institutions towards cellular manufacturing and away from drug development and purchase. How easy it will be to effect such a change remains to be seen

© 2003 Cancer Research UK

### S9

DENDRITIC CELL BIOLOGY AND POTENTIAL APPLICATIONS IN CANCER THERAPY.

Ingo Schmidt-Wolf, Medizinische Klinik und Poliklinik I, University of Bonn, Germany

Dendritic cells (DC) play a major role in the generation of immunity against tumour cells. DC can be grown under various culture conditions, which influence the phenotypical and functional properties of dendritic cells and there by the consecutive immune response mainly executed by T cells. Antigen receptors (TCR) of tumour-specific T cells recognise tumour-associated peptides that are presented in the context of HLA class-I or class-II molecules by the APC. Successful recognition of tumour-antigen by the T-cell is not only dependent on TCR-peptide-HLA-interaction, but other co-stimulatory signals must be provided to prevent anergy. These are mainly CD80/CD86-CD28- or CD40-CD40L-interactions. These interactions do not only underline the importance of T cells, but also the significant role of dendritic cells (DC), which are the most potent antigen-presenting cells among others like monocytes, macrophages and B cells. Several in vitro and in vivo studies showed the ability of vaccination with DC to elicit tumour-specific T-cell immunity. This result emplies that (1) DC might be just another altered element of the immune system or (2) DC are able to overcome tumour-protective alterations in cancer patients by inducing effective CTL response or (3) both. In this context a phenotypic and functional dichotomy of DC in DC1 and DC2 appears to be of importance. DC1and DC2 cells were found to produce different cytokines and there by induce TH1 and TH2 differentiation, respectively. The lymphoid-related DC (DC2) are CD11c$^{-}$ and have been shown to induce a tolerating response versus tumour cells by activating mainly TH2 cells, whereas myeloid-derived DC (DC1) are immunostimulatory via TH1 cells. Various conditions, which are important during generation and administration of dendritic cells to elicit a tumouricidal T cell-based immune response will be discussed.

### S10

CHROMOSOMAL TRANSLOCATIONS, FUSIONS GENES AND ACUTE LEUKAEMIA

Vaskar Saha, Cancer Research UK, Children's Cancer Group, London, Barts and The London E1M 7BQ

The acute leukaemias of childhood are characterised by specific non-random chromosomal translocations. These translocations occur as a result of chromosomal breakages and illegitimate repair and these events tend to occur early in life and often *inutero*. Translocation breakpoints occur at genomic hotspots and target genes, which are either transcription factors or tyrosinekinases. The genes appear to be part of a regulatory network that functions via chromatin mediated pathways in determining the development of a haematopoietic cell in a hierarchical fashion. Mostly translocations result in an in-frame fusion of the targeted genes to produce a chimaeric oncogene. It is thought that the chimaeric product dictates aberrant haematopoiesis and an arrest in differentiation. In parallel to the clinical situation, mice transgenic for these fusions develop leukaemia after a period of time. Moreover it is apparent that translocations can be detected in many more children than go on to develop disease. Thus while the fusion product initiates leukaemogenesis, other changes are necessary for disease to manifest. These secondary genetic events are currently not well characterised and transgenic models will play an important role in unraveling the sequence of events. An increase in the understanding of the nature of the fusion product and the regulatory pathways involved will in due course allow critical molecules to be targeted by novel therapeutic agents. Tyrosinekinase and histone deacetylase inhibitors are already in clinical trials and other molecules will follow. Within the understanding of leukaemogenesis lies a revolution in leukaemia therapy.

### S11

PHARMACOGENOMICS OF HUMAN CANCER: CHILDHOOD ACUTE LYMPHOBLASTIC LEUKEMIA (ALL) AS A PARADIGM

William E. Evans, on behalf of collaborators at St. Jude Children's Research Hospital, Memphis, TN, USA and Sophia Children's Hospital, Rotterdam, The Netherlands.

Acute lymphoblastic leukemia (ALL) comprises a number of genetic subtypes that are created by non-random chromosomal translocations producing aberrant gene fusions, with specific genetic subtypes having significantly different prognoses (Pui and Evans, *NEJM*, 1998). We have shown that the pattern of gene expression in ALL blasts, prior to treatment, discriminates among the major genetic and lineage subtypes of childhood ALL, and that gene expression profiles (U95A) are able to identify patients who have a high risk of treatment failure (Yeoh et al, *Cancer Cell*, 2002). More recently, we have taken a genome-wide approach (U95A) to determine whether there are non-random changes in gene expression following treatment with individual antileukemic agents or combinations, and whether gene expression differs according to the specific treatment given. This has revealed distinct treatment-specific changes in gene expression after *in vivo* exposure to anticancer agents, and established that leukemia cells of different genetic or lineage subtypes, share common pathways of genomic response to the same medications. Further, these studies have shown that treatment-induced changes in gene expression when the drug combination (HDMTX + MP) was given to treat ALL, differed markedly from the composite of the two agents when given alone (only 14% of genes that changed after single agents changed at the combination). To assess genomic determinants of the intracellular disposition of antileukemic agents, we have identified gene expression profiles (U95A) that discriminate the level of intracellular accumulation of methotrexate polyglutamates (MTXPG) after *in vivo* high-dose MTX treatment. Finally, we have used gene expression profiling (U133A) of leukemia cells to identify genes that discriminate ALL cells that exhibit *in vitro* sensitivity versus resistance (lowest third versus highest third for LC50 in MTT assay) to L-asparaginase, daunorubicin, and prednisolone, whereas vincristine sensitivity could not be discriminated. Collectively,these studies and those by others, illustrate the potential of gene expression profiling of primary cancer cells to illuminate genomic determinants of treatment response.

### S12

CML IN CHILDREN: MOLECULAR THERAPY VS MYELOABLATION

IRENE ROBERTS, IMPERIAL COLLEGE LONDON

Chronic myeloid leukaemia (CML) constitutes 2-3% of leukaemias in childhood with a prevalence of 1:100,000. The molecular basis, as in adults, involves a reciprocal translocation, t(9;22)(q34;q11), forming a BCR-ABL fusion gene & protein with constitutive tyrosine kinase activity. Management of childhood CML is directed by its triphasic nature: treatment response is optimal in chronic phase (CP) (median duration 4y); less good in accelerated phase (AP) (duration 6-12m); & poor in blast crisis (BC) (duration 3-6m). Current strategies for management require a choice between two radically different approaches and for children not only are the aims of treatment often different from adults, but there are also few data to inform this choice. The first option, stem cell transplantation (SCT) offers a high rate of long-term cure at the expense of significant mortality/morbidity. The second option, 'molecular' therapy with the tyrosine kinase inhibitor, imatinib, induces a high cytogenetic response rate with minimal morbidity but an unknown, and probably very low, chance of long-term cure. For children in CP the treatment of choice is SCT from an HLA-identical sibling donor (sibD) or fully matched volunteer unrelated donor (VUD). Recent data indicate overall 3-5y survival/predicted cure of >70% for sibD & 60% for VUD. Treatment of choice for children in AP or BC is SCT as soon as possible using the best available donor (mis-matched/haplo-identical donors if necessary) since the risk of treatment-resistance is high. Imatinib may be used in AP or myeloid BC to achieve temporary control, with SCT carried out as soon as evidence of imatinib-resistance is detected since this usually heralds rapidly transforming disease. This is an exciting time to be caring for children with CML. National studies of the natural history of childhood CML have been set up to investigate its aetiology in children & develop prognostic scores to guide therapy. Molecular monitoring post-SCT with early donor lymphocyte infusion (DLI) also offers the promise of improved long-term results & data from adult trials of imatinib & novel agents should help define the role of such innovative therapy in children.

© 2003 Cancer Research UK

**S13**

MRD IN CHILDHOOD ALL: HYPE OR HOPE.

Nick Goulden University of Bristol and Bristol Children's Hospital.

It is now more than a decade since techniques capable of detection of minimal residual disease (MRD) in childhood ALL were first described. Design flaws in retrospective studies dashed early optimism that clinical application of these techniques would lead to more accurate stratification of treatment. Gradually these problems have been overcome by technical innovation and the study of large homogeneous cohorts of patients with prolonged follow-up. There is now good evidence that clearance of MRD early in therapy is an independent prognostic factor within clinically homogeneous cohorts of children undergoing identical therapy. Consequently most of the major treatment consortia, including the UK Childhood Leukaemia working party have adopted MRD based stratification of therapy in frontline ALL protocols.

This presentation will focus on four main areas that must be addressed if the full potential of this technology is to be realised. First, it is important to note that the clinical significance of MRD at a given time point is dependent on the method employed to measure disease and the treatment protocol. Second, integration into clinical protocols requires standardised sampling, robust transport and quality assurance. Third as MRD technology is relatively expensive, and its benefit will only be seen after a number of years of follow up, long term funding will be required. Finally, it is vital that clinicians have realistic expectations of the benefits of MRD analysis. This is only one step in progress toward the ultimate therapeutic goal of cure of the greatest number of patients with minimal toxicity. Novel treatments tested in large international studies are also integral to this aim.

**S14**

TARGETING THE INVASIVE BEHAVIOUR OF MALIGNANT TUMOUR CELLS

Ian R Hart, Richard Dimbleby Department of Cancer Research/Cancer Research UK Laboratory, Rayne Institute, St. Thomas' Hospital, Lambeth Palace Road, London SE1 7EH.

Invasion, the violation of normal tissue boundaries as a consequence of deranged sorting of cell populations, is an integral component of the malignant phenotype. Presumably invasive behaviour up- or down-regulation of different genes, including those encoding cell adhesion receptors and proteolytic enzymes. To identify additional genes contributing to this phenotype we compared gene expression levels between the periphery and the centre of breast cancers using a combination of microdissection and microarray analysis. We showed that changes in gene expression, associated with minimal variation in microanatomical location of neoplastic cells, can be detected within even small tumour foci and that these fluctuations in gene expression levels contribute to invasive behaviour. While this search for novel genes involved in tumour progression is important we already know many of the "players" in this process. Polarised cell movement is a necessary component of tumour cell infiltration so that interference with the motility machinery could offer a way of significantly modifying invasive behaviour. Integrin hetrodimers, expressed at the tumour cell surface, play a major role in determining invasion. Thus we have shown that the C-terminal 11 amino acids of the β6-subunit are essential for mediation of αvβ6-dependent invasion by oral squamous cell carcinomas. Abrogation of invasive activity of such tumour cells was achieved by intracellular delivery of the corresponding sequence of 11aa, either as membrane-permeable chimeric peptides or introduced as a minigene by retroviral transduction. Similar changes in human breast cancer cell migration epidermal growth factor (EGF)-induced chemotaxis, were achieved using comparable techniques to target the Protein kinase Cα(PKCα)-binding sequence of the β1-integrin subunit. Together these findings demonstrate that the exogenous, or retrovirally-mediated, delivery of peptides targeted to specific regions of integrin cytoplasmic tails may provide a novel method for developing anti-invasive therapies.

These studies also suggest that identification of alterations in integrin levels, an understanding of the irinteractions with co-localising proteins and the targeting of specific sites within these molecules all offer ways of reducing the potential of malignant cells to violate normal tissue boundaries.

**S15**

TRANSCRIPTION OF NEURONAL GENES IN LUNG CANCER

Judy M. Coulson*, Physiological Laboratory, The University of Liverpool

Lung cancer continues to be the commonest fatal malignancy in the developed world and is the biggest cancer killer in the UK. Lung cancers are broadly classified as small cell lung cancer (SCLC) or non-small cell lung cancer (NSCLC). SCLC is aggressive in nature and accounts for around 20% of lung cancers, it is distinguished from NSCLC by expression of both epithelial and neuroendocrine markers, including many neuropeptides. Our studies into transcriptional regulation of the neuropeptide arginine vasopressin (AVP) in SCLC identified several regulatory motifs in the proximal promoter that bind two classes of transcription factor, neurone restrictive silencer factor (NRSF/REST) and upstream stimulatory factors (USF-1 & USF-2) (reviewed in Coulson, 2002, Prog Brain Res, 139: 329). such transcription factors and the downstream genes they regulate may represent valuable SCLC-specific markers or therapeutic targets. Although USF factors were regarded as ubiquitous, we have shown differential expression of USF-2 in lung cancer types and overexpression of USF-2 in NSCLC activated the AVP promoter reporter gene constructs via canonical and non-canonical E-box motifs (Coulson et al, 2003, Biochem J, 369, 549). Other gene promoters with USF binding sites show similar regulation patterns in lung cancer cell lines. In contrast, the transcriptional repressor NRSF was regarded as restricted to non-neuronal cells, but isoforms have recently been identified in both neuronal cells and in SCLC (Coulson et al, 2000, Cancer Res, 60, 1840). Experimental evidence suggests that NRSF isoforms dysregulate repressor function and we have shown that several NRSE-regulated genes are differentially expressed in SCLC and NSCLC. Our current research program includes targeting NRSF isoforms with inhibitory RNA; candidate gene, proteomic and DNA microarray analyses; and validation in clinical samples.

**S16**

GENE ICE AS APPLIED TO STUDIES OF HORMONALLY-RESPONSIVE CANCERS

Danish Mazhar*, Simak Ali, Laki Buluwela, Jonathan Waxman and R. Charles Coombes, Dept of Clinical Oncology, Hammersmith Hospital, Ducane road, London W12 OHS

Breast cancer growth is dependent on oestrogens acting via the oestrogen receptor (ER). Prostate cancer provides an equivalent model of hormone-responsive malignancy in men, with growth of tumours being driven by androgens acting at the androgenreceptor (AR). Treatment of both diseases is limited by the development of hormone independence. A novel strategy for silencing genes by targeting histone deacetylation has been developed. In the case of ER- and AR-regulated genes, this has been achieved by fusing ER and AR with the transcriptional repressor PLZF to create the hybrid molecules PLZF-ER and PLZF-AR. Specific repression of both reporter and genomically encoded genes has been shown. This technology, termed Gene Inactivation by Chromatin Engineering ("GeneICE"), provides a molecular form of "anti-oestrogen or -androgen" genetherapy, with the potential advantage of switching off ER- andAR-regulated genes in an irreversible way, overcoming the problem of acquired "resistance".

We have shown that PLZF-ER, when stably transfected into the ER-expressing cell line, MCF-7, causes repression of endogenous oestrogen-regulated gene expression and cell growth arrest inculture. In female nude mice implanted with 60-day release oestradiol pellets, subcutaneous injection of MCF-7 cells transfected with PLZF-ER resulted in no tumour formation in 12/12 compared with 6/12 mice in the control group injected with the parent MCF-7 Tet Off cell line. In similar studies, PLZF-AR has been tranduced into the androgen-responsive prostate cancer cell line, LNCaP. The resulting cells show an inhibition of androgen-regulatedexpression of Prostate Serum Antigen (PSA).

These results provide evidence ofthe potential of Gene ICE to silence ER- and AR-regulated genes in a specific manner. Moreover, it has been shown that breast cancer cell growth in vitro and in vivo can be potently inhibited. We believe that this technology could be used to develop new therapies in breast and prostate cancer.

© 2003 Cancer Research UK

### S17

CELLULAR SENESCENCE -- IMPLICATIONS FOR CANCER PROGRESSION

Judith Campisi, Lawrence Berkeley National Laboratory, 1 Cyclotron Road, Berkeley, CA 94720 USA; and Buck Institute for Age Research, 8001 Redwood Blvd., Novato, CA 94945

Normal cells can respond to potential cancer-causing stimuli by undergoing cellular senescence, a state characterized by an essentially irreversible arrest of cell proliferation and often striking changes in cellular function. Several lines of evidence support the idea that the senescence response is an important tumor suppressor mechanism in mammals. Recent evidence, however, suggests that cellular senescence is also an example of evolutionary antagonistic pleiotropy. Accordingly, these data suggest that while cellular senescence protects organisms from cancer early in life, later in life it can promote aging phenotypes, including late life cancer. We have found that senescent human stromal cells can promote the neoplastic progression of premalignant epithelial cells in culture and *in vivo*. These findings raise the possibility of developing novel strategies for cancer prevention -- strategies aimed a eliminating or reversing the deleterious effects of senescent cells. We are currently determining the molecular pathways by which senescent cells acquire their altered pro-carcinogenic phenotypes, and developing tools for reversing the senescent phenotype and/or selectively eliminating senescent cells.

### S18

TELOMERASE, CELL MORTALITY AND GRN163, A POTENT AND SPECIFIC TELOMERASE INHIBITOR FOR CANCER THERAPY

Calvin B. Harley, Geron Corporation, Menlo Park, CA, 94025

Telomerase is an enzyme required for maintenance of telomeric DNA "capping" the ends of eukaryotic chromosomes. It is a unique ribonucleoprotein reverse transcriptase for which no redundant enzyme nor efficient alternative pathway is known. Without telomerase, telomeres shorten to a critical length, chromosomes become unstable, and cell cycle arrest or death ensues. Human telomerase is tightly repressed in most normal somatic cells, transiently inducible in certain stem or progenitor cells, and constitutively activated in germline and tumor cells. Multiple academic groups and biotechnology and pharmaceutical companies have targeted telomeres or telomerase for potential therapeutic products over the past 5-10 years. Although there are thousands of publications, including a number of positive cell- and animal-based efficacy studies, currently there are only a small number of drug candidates in development. The first clinical studies based upon telomerase target the catalytic protein component (hTERT) as an antigen for therapeutic vaccines against cancer, and show preliminary safety and some signs of efficacy. We are now in IND-enabling development of GRN163, a highly potent and specific N3'-P5' thio-phosphoramidate oligonucleotide inhibitor of telomerase. GRN163 tightly binds to the template region of the RNA component of telomerase, hTR, blocking the active site of the enzyme. GRN163 inhibits telomerase activity and prevents tumor cell growth in culture and *in vivo* in multiple model systems, with few if any signs of toxicity to normal cells or tissue. The effects of GRN163 on tumor growth are generally more rapid in cancer cells with short telomeres, and may be augmented when used in conjunction with cytotoxic or DNA damaging agents. In conclusion, the data suggest that GRN163 has the potential to be a universal anti-cancer agent with acceptable toxicity.

### S19

G-QUADRUPLEX FORMATION AT TELOMERE ENDS: A STRATEGY FOR SELECTIVE INTERFERENCE WITH TELOMERE MAINTENANCE IN TUMOUR CELLS

Stephen Neidle, *Cancer Research UK Biomolecular Structure Group The School of Pharmacy, University of London, UK*

The G-rich telomeric DNA sequences of eukaryotic chromosomes are involved in a nucleoprotein complex that protects these ends from degradation and recombination. They are also maintained in length in almost all cancer cells by the action of the reverse transcriptase enzyme telomerase, whose catalytic sub-unit is not expressed in normal somatic cells. Telomerase catalyses the addition of further telomeric DNA repeats onto the extreme 3' end of the telomere, and also caps the end. Inhibition of the catalytic function of telomerase has been shown to result in progressive telomere shortening, leading to eventual replicative senescence and cell death. However this requires a long lag time, dependent on initial telomere length. An alternative strategy focuses on telomeric DNA, and uses small molecules to induce it to fold into higher-order G-quadruplex structures. We suggest that this has a dual effect, of (i) inhibiting the catalytic activity of telomerase, and (ii) hindering telomerase and other proteins from capping and protecting the 3' ends, thus enabling the rapid onset of senescence.

A family of tri-substituted acridine-based small molecule G-quadruplex interactive molecules, have been developed by us, utilising structure-based design principles. The most active molecules in this series inhibit telomerase at 18nM and result in telomere shortening in several tumour cell lines after long-term treatment. They also rapidly produce senescence and demonstrate anti-cancer activity *in vivo* with cell lines and tumour models that have relatively short telomeres at the outset. The presentation will review the quadruplex approach, and discuss its therapeutic potential.

### S20

TELOMERASE-DIRECTED GENE THERAPY

W. Nicol Keith, Cancer Research UK Department of Medical Oncology, University of Glasgow, Cancer Research UK Beatson Laboratories, Glasgow G61 1BD, UK. n.keith@beatson.gla.ac.uk

Suicide gene therapy aims to selectively target cancer cell without harming normal cells thus reducing the toxicity often associated with conventional therapies. In order to achieve this selectivity, gene therapy approaches require mechanisms to regulate and limit the expression of therapeutic genes to cancer cells. Achieving this aim remains a challenge for the development of clinically useful suicide gene therapy. Tumour-specific gene promoters can be used for transcriptional targeting to improve selectivity and increase therapeutic index. However until recently this has been difficult and disappointing as many tumour specific promoters show weak transcriptional activity and are unable to drive efficient expression of the therapeutic gene. In addition, the promoter activity is often restricted to one tumour type for example HER2/NEU positive is limited to use with breast cancer. As a solution we have developed the telomerase hTR and hTERT promoters as highly efficient, cancer specific transcriptional regulators capable of targeting suicide gene therapy constructs to a broad range of cancer types. Proof of concept has been achieved using telomerase targeted adenoviral suicide gene therapy vectors, using the promoter sequences to regulate expression of the bacterial NTR gene within a replication defective adenoviral delivery system. *In-vitro / In-vivo* data demonstrates the benefits of these telomerase vectors to effectively regulate the expression of genes to activate cytotoxic compounds in cancer cells and not normal cells. In all our studies the hTR promoter out performs the hTERT promoter suggesting that the hTR is the preferred candidate for therapeutic development. Web Review: Telomerase-Directed Molecular Therapeutics. Expert Reviews in Molecular Medicine, http://www.expertreviews.org/02004507h.htm

© 2003 Cancer Research UK

### S21

**ABSTRACT NOT RECEIVED**

### S22

TARGETED RADIOTHERAPY OF BRAIN TUMOURS
Michael R. Zalutsky, Department of Radiology, Duke University Medical Center, Durham, NC USA.

External beam radiation therapy is rarely curative for gliomas and other central nervous system malignancies due to its lack of tumor specificity. Moreover, external beam radiation generally results in damage to adjacent normal tissues, compromising neurologic function and quality of life in the few patients who do survive. Targeted radiotherapy is an attractive alternative treatment strategy that utilizes a molecular vehicle such as a monoclonal antibody (mAb) to selectively deliver radionuclides to tumour cells. Radionuclides that emit α-particles such as 7.2-h half-life $^{211}$At (astatine) offer the possibility of combining cell-specific molecular targeting with radiation having range of only a few cell diameters. Furthermore, α-particles are considerably more cytotoxic that conventional radiation and their effectiveness is independent of dose rate and oxygen. Currently, we are performing a Phase I trial evaluating $^{211}$At-labeled human-mouse chimeric anti-tenascin 81C6 mAb administered into surgically created tumour resection cavities in recurrent glioma patients. Astatine-211 was produced at the Duke University Medical Center cyclotron and mAb 81C6 was labeled with preservation of immunoreactivity using *N*-succinimidyl 3-[$^{211}$At]astatobenzoate. Seventeen patients including 13 with glioblastoma multiforme received 10 mg of mAb labeled with escalating activities (2-10 mCi) of $^{211}$At. Retention of activity in the resection cavity was excellent with less than 0.2% of the injected dose found in the blood pool. Median survival in these recurrent brain tumour patients was 60 weeks, and 3 patients including 2 with glioblastoma multiforme survived for 3 years. We are currently performing preclinical studies to evaluate the potential of other $^{211}$At-labeled compounds for the targeted radiotherapy of brain tumors.

### S23

ONCOLYTIC VIRUS THERAPY FOR BRAIN TUMOURS
Roy Rampling
Professor of Neuro-oncology, University of Glasgow

Oncolytic viruses have the ability selectively to replicate in cancercells, thereby causing cell lysis and death. They do not rely onadditional agents to effect tumour kill, as in ‘suicide gene therapy’. Such selectivity has required genetic modification to existing ‘wild-type’ viruses to render them incapable of replication in normal cells. Early examples of such agents frequently involved gene substitutionor deletion. They included adenovirus (e.g. Onyx 015), Poliovirus(PV1(RIP0)), and Herpes virus (HSV1716). Newer agents such as thegene enhanced adenovirus Delta-24 RGD are showing increasing promise.

Malignant gliomas have several potential advantages for oncolyticviral development. They are differentiated from the brain by activereplication, and the major gene changes that drive this growth andreplication are increasingly understood. Major disadvantages howeverare the inherent heterogeneity of gliomas and the difficulties ofdelivery of agents to the brain.

Oncolytic viruses have been evaluated in early clinical trial usingsimple direct injection techniques (e.g HSV1716, E1B-). Whilst thesehave been shown to be safe, efficacy data beyond anecdotal reports arelacking. Neither has it been possible to establish clearly the optimalapplication dose for an agent that is potentially self-replicating.HSV 1716 has been shown to be safe when injected directly intotumour and in brain adjacent to tumour in doses up to 106 pfu.Furthermore, examination of tumour following resection has shownevidence of viral replication. Plans for a formal efficacy evaluation arewell advanced. These results and issues of trial design will bediscussed in detail.

We have demonstrated synergy when HSV 1716 is used inconjunction with radiation to kill tumour cells. We also have indevelopment second generation viruses using the basic HSV1716structure to deliver transgenes to enhance tumour kill using a ‘suicide’mechanism. These results will be discussed as will the potential for‘continuous enhanced diffusion’ to improve delivery. Oncolytic viraltherapy shows promise in the management of malignant glioma.

### S24

GENETIC ABNORMALITIES ASSOCIATED WITH ASTROCYTOMA PROGRESSION
V. P. Collins, Koichi Ichimura, Lu Liu, Magnus Backlund
Department of Pathology, University of Cambridge, UK

Astrocytic tumours have been known to progresswith time to moremalignanttumour forms for over a century. We have been studying 190 astrocytic gliomas (136 glioblastomas (GB), 39 anaplastic astrocytomas (AA) and 15 astrocytomas (A)) for abnormalities of genes in the RB1pathway (*CDKN2A*, *CDKN2B*, *CDK4* and *RB1*), the p53 pathway (*p14*$^{ARF}$, *MDM2*, and *TP53*), as well as *PTEN* and *EGFR*. A and AA hadno wild-type *TP53* or one mutated allele in 67% of cases. Only29% ofthe GB had no wild type *TP53*with an additional 6% having one mutatedallele. Wild type *p14ARF* was absent from 38% of GB and a further 8%had amplification and overexpression of *MDM2*. Thus 76% of GB (103/136), 72% of AA (28/39) and 67% of A (10/15) had a deregulatedp 53 pathway indicating that this is almost a prerequisite for astrocytictumors. Abnormalities of the RB1 pathway occurredin 21% AA and 67% GB either by mutation/homozygous deletion of *RB1*, *CDKN2A* and*CDKN 2B*, or amplification of *CDK4*. This indicates that disruption of the RB1 pathway is directly related to as trocytic tumour progression. Amplification of the *EGFR* gene was not observed in A, was unusual in AA (8%) but common in GB (33%). Loss of wild type *PTEN* occurred in one AA (3%) but was very common in GB (47%) and could be found together with all combinations of the other genetic abnormalities. Survival of patients with GB is typically less than one year, We studied whether any of the genetic factors listed above were related to survival inGB alone. We found that abnormalities in any of the four genes (*CDKN2A*, *CDKN2B*, *RB1*, *CDK4*) codingfor components of the Rb1 pathway were associated with shorter survival (p=0.002). In combination with loss of wild-type *PTEN* the association was even stronger (p<0.001), the median post-operative survival being 166 days as compared with thegroupwithout these abnormalities where the median survival was 437 days. The survival difference remained statistically significant in Cox Regression analysis adjusting for age (p=0.012). The findings indicate that knowledge of the molecular genetic abnormalities in glioblastomas provides important data in assessing individual patients. As further advances in our understanding of the molecular genetics and cell biologyof gliomas are made, in addition to providing prognostic information, such data may also provide targets for innovative therapy in the individual case.

© 2003 Cancer Research UK

**S25**
REGULATION AND FUNCTION OF THE P53 TUMOUR SUPPRESSOR PROTEIN
Karen Vousden, Beatson Institute for Cancer Research, Switchback Road, Bearsden, Glasgow G61 1BD, UK

p53 plays an important role in preventing tumour development by responding to stress signals that are encountered during malignant progression. Several responses to p53 have been described, including cell cycle arrest, senescence, differentiation and apoptosis. Cell type, cell environment and presence of cooperating signals can all contribute to the choice of response. Also, the ability to induce cell cycle arrest and apoptosis are separable functions of p53 that can be controlled independently. p53-induced apoptosis is important for tumour suppression, and virtually all cancer cells have acquired mechanisms to evade this response. In many cancers this is achieved by mutation of p53 itself, but tumours that retain wild type p53 can show defects in the ability to activate p53. Stress-induced activation of p53 involves stabilization of the p53 protein by inhibition of MDM2, the ubiquitin-ligase responsible for targeting p53 for degradation. Several mechanisms for the inhibition of MDM2 have been described, including down-regulation of MDM2 expression, phosphorylation of p53 or MDM2 and the interaction of MDM2 with small proteins such as $p14^{ARF}$. In some cancers failure to activate p53 is associated with defects in the ability to inactivate MDM2, suggesting that MDM2 inhibitors may allow reactivation of p53 in this class of cancers. We have developed a high throughput screen to identify small molecules that will inhibit MDM2 to allow stabilization and activation of p53 and so, to some extent, mimic the function of proteins like $p14^{ARF}$. A group of related small molecules identified in this screen may have utility as therapeutic agents.

**S26**
NEW AGENTS: NEW TRIALS; ARE THE RULES OR EXPECTATIONS CHANGING?

Dr George Blackledge, VP, Medical Director of Oncology
AstraZeneca, Alderley Park, Macclesfield

There has been an explosion in the number of new potential anti-cancer targets developed over the past few years and in therapeutic modalities directed against them. The specificity of these targets, and the number of tumours and patients in whom they are a primary cause of cancer drive has meant that both pre-clinical and clinical assessment has been different from conventional anti-cancer chemotherapy drug development.

There is now an emerging body of data concerning the new targetted agents. These agents tend to have a relatively low response rate of between 10-20% (there are a few notable exceptions to these where a single mutated gene is targetted); they have demonstrated activity in advanced disease and evidence of clinical benefit as judged by disease symptom improvement, but with one or two exceptions their activity in combination with standard cytotoxic chemotherapy has been disappointing.

These observations raise important questions about the future of oncology drug development with targetted agents. Trial design needs to be tailored to expectations of outcome and patient or tumour selection becomes ever more important for the optimal use of such agents. These new targetted agents do represent a genuinely novel and potentially important development for cancer treatment and therefore expectations should be managed with these agents and the way in which clinical trials are carried out should reflect the available data.

**S27**
DEVELOPING DRUGS FOR CANCER: STRIKING BALANCE BETWEEN CLINICAL PRAGMATISM AND SCIENTIFIC ALLURE
Hilary Calvert, Northern Institute for Cancer Research, University of Newcastle upon Tyne

During the last decade Phase III randomised trials have demonstrated survival improvements in several common malignancies resulting from the use of new cytotoxic drugs, examples being taxanes in breast and ovarian cancer, irinotecan in colorectal cancer and gemcitabine in lung cancer. During the same period the identification of new targets as a result of advances in understanding the molecular pathology of tumours, coupled with advances in medicinal chemistry and antibody technology, has resulted in the availability of a increasing number of “targeted” agents interacting with processes controlling tumour growth and cell division. Although some of these, for example trastuzumab and imatinib, have been spectacularly successful, others have produced disappointing Phase III data. It is accepted that a number abnormalities of growth control processes are required to generate most of the common malignancies so perhaps not surprising that a single therapeutic agent working very specifically on a single target may have a minimal effect. If we can develop the ability to define the lesions driving a particular patient’s tumour we would be in a position to select an individualised combination appropriate for that patient. Future treatments are likely to be multimodal, utilising traditional cytotoxics and targeted agents. Techniques for treatment individualisation can be applied to cytotoxic agents since many of these also have well defined cellular targets, blurring the distinction between the two. One multimodal approach being developed in the Northern Institute for Cancer Research is to develop inhibitors of DNA repair, specifically of poly(ADP-ribose) polymerase (PARP) and DNA protein kinase for use in conjunction with chemotherapy and radiotherapy. A Phase I trial ofa PARPinhibitor is currently under way.

**S28**
IS NEOADJUVANT CHEMOTHERAPY OF BENEFIT IN THE TREATMENT OF LOCALLY ADVANCED CERVICAL CANCER? JF Tierney MRC Clinical Trials Unit, 222 Euston Road, London NW1 2DA, UK.

Despite the enrolment of more than 3000 women in randomised trials, the benefits and risks of neoadjuvant chemotherapy in the treatment of locally advanced cervical cancer remained uncertain. We carried out a systematic review and meta-analysis of individual patient data to assess its effect in two comparisons. The first comparison was of neoadjuvant chemotherapy followed by radical radiotherapy compared to the same radiotherapy alone. When all 18 trials were considered together, a high level of statistical heterogeneity suggested that the results could not be combined indiscriminately. Much of the heterogeneity was explained by separate analyses of groups of trials. Trials using chemotherapy cycle lengths shorter than 14 days (HR=0.83, p=0.046) or cisplatin dose intensities greater than $25mg/m^2$per week (HR=0.91, p=0.20) tended to show an advantage for neoadjuvant chemotherapy on survival. In contrast, trials using cycle lengths longer than 14 days (HR=1.25, p=0.005) or cisplatin dose intensities lower than $25mg/m^2$ per week (HR=1.35, p=0.002) tended to show a detrimental effect of neoadjuvant chemotherapy on survival. The second comparison was of neoadjuvant chemotherapy followed by surgery compared to radical radiotherapy alone. The combined results from 5 trials (HR=0.65, p=0.0004) indicated a highly significant reduction in the risk of death with neoadjuvant chemotherapy, but there were some differences between trials in their design and results. Despite some unexplained heterogeneity, the timing and dose intensity of cisplatin-based neoadjuvant chemotherapy appears to have an important impact on whether or not it benefits women with locally advanced cervical cancer and warrants further exploration.

© 2003 Cancer Research UK

### S29

1ST-LINE CHEMOTHERAPY FOR OVARIAN CANCER – CURRENT STATUS

A. du Bois, Dr.-Horst-Schmidt-Kliniken, Wiesbaden, Germany

Platinum-based chemotherapy is the mainstayof systemic treatment of advanced ovarian cancer. However, details are at least partially contro-versial. Among others, the question regarding the impact of combination regimens compared to single agent platinum is cause for vital discussion. Until now, no randomised trial has ever shown superiority over single agent carboplatin. However, 2 randomised trials have shown superiority for cisplatin-paclitaxel (PT) over cisplatin-cyclophosphamide (PC), a regimen formerly believed to be at least equivalent with carboplatin. The average survival gain in these trials was about 1year (HR 0.7 and 0.75). 3 further trials compared PT versus carboplatin-paclitaxel (TC) showing at least comparable activity and better tolerance and quality of life for the latter. Based on these results, TC was adopted as new standard arm in almost all study groups and randomised trials. Questions about the role of platinum-paclitaxel combination chemotherapy have been raised when ICON-3 reported no superiorityfor TC compared to single agent carbo-platin or PAC and GOG # 132 failed to show superiority of PT over single agent cisplatin. Both trials are difficult to bring in line with GOG # 111 and OV-10. However, both trials reported a considerable cross-over rate in the single agent platinum arm (GOG#132 > 50% even before progression; ICON-3 about 40% mainly after progression). Furthermore, the designof ICON-3 makes it difficult to compare it with other trials (more heterogenous population including FIGO I pts., individual choice of standard arm, different randomisation procedures, differences with respect to participating countries / studygroups / recruitment numbers per centre, no audit or monitoring in contrast to GOG # 111 and OV-10). Moreover, response rates in ICON-3 were not reported, neither was a comparison performed between comparable subgroups among GOG 111, OV-10,and ICON-3 , although data have been merged for a meta-analysis. Last but not least, surgical details of ICON-3 can only be interpreted based on the publication and show a rather low percentage of completely debulked patients. In summary, neither ICON-3 nor GOG132 have shown superiority of any regimen compared to PT/TC, and the meta-analysis of all 4 trials did still show benefit for TC. Considering all 4 trials, it cannot be ruled out that at least some pts. would benefit from combination therapy. Therefore, TC remained the standard arm as chosen by all study groups (including the ICON group). Outside of trials, single agent carbo might be an option for pts. not willing to trade a possible benefit for the additional taxan associated toxicity – with the option of receiving paclitaxel in case of relapse or persisting tumor.

### S31

OVARIAN CANCER: MOLECULAR STRATEGIES FOR A LOCOREGIONAL PROBLEM

Hani Gabra
Cancer Research UK, Edinburgh Cancer Research Centre, Crewe Road South, Edinburgh, UK

The natural history of ovarian cancer is characterised by progression of bulky loco-regional peritoneal dissemination in the relative absence of visceral metastatic disease. Most patients die from this dominant clinical scenario. An early lesion for epithelial ovarian cancer has not been defined, and there is no strong evidence that early stage ovarian cancer commonly progresses to advanced stages. These uncertainties coupled with low prevalence of ovarian cancer in the population present intellectual and practical difficulties for screening of the disease. Strategies that re-focus on control of peritoneal dissemination of ovarian cancer rather than either screening or total eradication of systemic disease might therefore be expected to improve prognosis. Our understanding of the relationship between ovarian cancer and the peritoneum is poor. There are some notable examples of progress, however. In this talk I will review the relationships between ovarian epithelial oncogenesis, inflammation, cellular adhesion and cell death. Evidence from our own work and that of others on molecules involved in these processes will be presented in order to develop consistent testable hypotheses for molecular interventions to control progressive peritoneal dissemination of ovarian cancer.

### S30

PROGRESS IN SCREENING FOR OVARIAN CANCER

Ian Jacobs, Bart's and the London, Queen Mary School of Medicine, London, UK

Efforts to screen for early OC are based upon the hypothesis that outcomes will improve if OC is diagnosed and treated whilst asymptomatic and preclinical. There is good evidence from large prospective population studies of over 100,000 women that preclinical OC can be detected by transvaginal ultrasound or by a multimodal approach using CA 125 as a 1$^{o}$ test followed by ultrasound as the 2$^{o}$ test in selected cases. The available data suggest that the multimodal approach has a lower false positive rate, whilst the ultrasound approach may have higher sensitivity. The sensitivity of the multimodal approach has been improved by use of 'Risk of Ovarian Cancer' (ROC) algorithm to interpret CA125 results which assesses the pattern of CA 125 over time rather than in relation to a simple cut off.

On the basis of this progress a major national three arm randomised control trial involving 200,000 postmenopausal women, called UKCTOCS (UK Collaborative Trial of Ovarian Cancer Screening) was launched in 2001. The study involves randomisation to a control arm (100,000), a multimodal arm screened with the ROC algorithm followed by ultrasound (50,000) and a transvaginal ultrasound arm (50,000). The primary end point for the study is the impact of screening on ovarian cancer mortality. In addition UKCTOCS will assess the issues of target population, compliance, health economics as well as the physical and psychological morbidity of screening. A second study is underway to optimise screening in women at high risk of OC because of a strong family history of cancer (UKFOCSS – UK Familial Ovarian Cancer Screening Study). When data from these studies becomes available during the next decade the place of OC screening in both the high risk and general populations should be clarified.

© 2003 Cancer Research UK

